# Draft Genome Sequences of Two *Propionibacterium acnes* Strains Isolated from Progressive Macular Hypomelanosis Lesions of Human Skin

**DOI:** 10.1128/genomeA.01250-15

**Published:** 2015-11-05

**Authors:** Rolf Petersen, Hans B. Lomholt, Christian F. P. Scholz, Holger Brüggemann

**Affiliations:** Department of Biomedicine, Aarhus University, Aarhus, Denmark

## Abstract

*Propionibacterium acnes* is a Gram-positive bacterium that is prevalent on human skin. It has been associated with skin disorders such as acne vulgaris and progressive macular hypomelanosis (PMH). Here, we report draft genome sequences of two type III *P. acnes* strains, PMH5 and PMH7, isolated from PMH skin lesions.

## GENOME ANNOUNCEMENT

*Propionibacterium acnes* is a Gram-positive bacterium constituting a significant part of the human skin microbiota. It has been associated with skin diseases such as acne vulgaris, and with nonskin diseases such as sarcoidosis as well as medical device-related and postoperative infections (1). Using multi and single-locus sequence typing (MLST/SLST) schemes, the *P. acnes* species has been subdivided into the phylogenetic types I, II, and III, and several subtypes (2).

Here, we present the genome sequences of two type III strains, designated PMH5 and PMH7. Both strains were isolated from patients in Aalborg, Denmark, suffering from progressive macular hypomelanosis (PMH), a skin disorder characterized by hypopigmented macules distributed mainly on the trunk and usually seen in young adults (3). The full study protocol and all procedures were approved by the Ethics Committee of Denmark Region North under file N-20120050. After swab sampling from PMH lesions present on the lower back of two patients, the *P. acnes* isolates were cultivated on Brucella agar plates. SLST (4) revealed their type III identity, and microscopy confirmed the previously described distinct morphological appearance of type III strains compared with type I and type II strains (5).

Genomic DNA of *P. acnes* was isolated using the MasterPure Gram-positive DNA purification kit (Epicentre). A genomic library was constructed and subjected to paired-end sequencing using a HiSeq Illumina sequencer at the Beijing Genomics Institute (Shenzhen, China). The assembly of sequence reads was done using SOAPdenovo (version 1.05); it resulted in draft genomes of 2,566,561 bp (71 contigs in 15 scaffolds) and 2,567,819 bp (47 contigs in 13 scaffolds) for strains PMH5 and PMH7, respectively. The G+C content was 60% for both draft genomes. The Prokaryotic Genome Automatic Annotation Pipeline (PGAAP) of NCBI predicted 2,372 and 2,375 genes in strains PMH5 and PMH7, respectively.

Genome information about type III *P. acnes* is scarce. This study doubles the number of available type III genomes. The other available type III genomes are from *P. acnes* strains HL201PA1 (6) and JCM18909 (GenBank accession numbers [and lengths]: AODA01000000 [2,563,562 bp] and BAVM01000000 [2,573,824 bp], respectively). *P. acnes* type III strains differ in several genomic regions from the genomes of other *P. acnes* types; based on a bidirectional BLAST analysis, approximately 20% of the identified genes are unique to type III genomes compared to genomes of types I and II.

The frequency of type III strains on human skin and their potential involvement in PMH remain open future research questions. The genome sequences of the two type III strains of *P. acnes* will also provide a valuable resource for (comparative) skin microbiota studies.

### Nucleotide sequence accession numbers.

The draft genome sequences were deposited in the DDBJ/EMBL/GenBank database under the accession numbers LJAS00000000 (*P. acnes* PMH5) and LJAT00000000 (*P. acnes* PMH7).
